# Delinking resident duty hours from patient safety

**DOI:** 10.1186/1472-6920-14-S1-S2

**Published:** 2014-12-11

**Authors:** Roisin Osborne, Christopher S Parshuram

**Affiliations:** 1 Center for Safety Research, Hospital for Sick Children, Toronto, Ontario, Canada; 2 Child Health and Evaluation Sciences Program, The Research Institute, Hospital for Sick Children, Toronto, Ontario, Canada; 3 Centre for Patient Safety, University of Toronto, Toronto, Ontario, Canada; 4 Institute of Medical Sciences, University of Toronto, Toronto, Ontario, Canada; 5 Department of Critical Care Medicine, Hospital for Sick Children, Toronto, Ontario, Canada; 6 Department of Paediatrics, Hospital for Sick Children, Toronto, Ontario, Canada; 7 Department of Paediatrics, University of Toronto, Toronto, Ontario, Canada; 8 Interdepartmental Division of Critical Care Medicine, University of Toronto, Toronto, Ontario, Canada; 9 Institute of Health Policy, Management and Evaluation, University of Toronto, Toronto, Ontario, Canada

## Abstract

Patient safety is a powerful motivating force for change in modern medicine, and is often cited as a rationale for reducing resident duty hours. However, current data suggest that resident duty hours are not significantly linked to important patient outcomes. We performed a narrative review and identified four potential explanations for these findings. First, we question the relevance of resident fatigue in the creation of harmful errors. Second, we discuss factors, including workload, experience, and individual characteristics, that may be more important determinants of resident fatigue than are duty hours. Third, we describe potential adverse effects that may arise from – and, therefore, counterbalance any potential benefits of – duty hour reductions. Fourth, we explore factors that may mitigate any risks to patient safety associated with using the services of resident trainees.

In summary, it may be inappropriate to justify a reduction in working hours on the grounds of a presumed linkage between patient safety and resident duty hours. Better understanding of resident-related factors associated with patient safety will be essential if improvements in important patient safety outcomes are to be realized through resident-focused strategies.

## Introduction

The ideal resident duty schedule to maximize patient safety has not yet been identified. In fact, the notion of an ideal schedule may be too simplistic given the diversity of residency programs and training requirements, variations in clinical workload, and differences between individuals with respect to personal preferences and tolerance of fatigue. As such, the creation of a resident duty schedule that maximizes patient safety may be an inappropriate, albeit well-intentioned, aspiration.

In this narrative review we focus on the relationship between resident duty hours and patient safety. We describe the well-recognized relationship between fatigue and error, and the seemingly contradictory evidence that suggests that a reduction in the number of duty hours is not associated with improved patient safety. Next, we explore four possible reasons why the literature has not confirmed the popular expectation that shorter duty hours improves patient safety. First, it is possible that the fatigue arising from resident duty hours is a relatively minor determinant of significant medical error. Second, duty hours may be only a minor factor contributing to resident fatigue. Third, it is possible that the adverse consequences of duty hour reduction will counterbalance any beneficial effects of reduced fatigue. Fourth, the service provided by residents may be of limited consequence to patient safety. While there may be other scientifically or socially valid reasons for duty hour reduction, these are outside the scope of this review.

## The relationship between resident duty hours and patient safety

When evaluating research describing resident duty hours and patient safety, one must carefully consider study design and the patient safety outcome(s) presented. It is important to separate intermediate outcomes such as potential errors, errors without clinical consequence, and perceptions of safety from definitive patient outcomes such as harmful errors, preventable harm, mortality rates, and risk-adjusted mortality rates.

The greatest volume of evidence linking prolonged resident duty hours to compromised patient safety derives from the laboratory-based evaluation of sleep deprivation and performance. This includes the popular work by Dawson and Reid [1] and other studies that align the effects of sleep deprivation with that of alcohol ingestion [2,3]. Other laboratory work suggests that progressive increases in sleep deprivation are associated with slower reaction times and decreased performance on other tests. A meta-analysis of 60 studies on sleep deprivation (with a total sample of 959 resident physicians and 1,028 non-physicians) evaluated performance in resident physicians and found a 1.5 standard deviation reduction in performance in a wide variety of tests after less than 30 hours of continuous wakefulness. This review found greater effects of sleep deprivation in non-physicians as compared with resident physicians. The authors attributed the differences between resident physicians and non-physicians to chronic sleep deprivation in the resident controls and to differences in the amount of sleep before the study period [4]. Studies using self-reported and objective measures of residents’ sleep confirm that acute sleep deprivation is routine, but question the frequency of chronic sleep deprivation and suggest that on-call residents do sleep while they are on duty [5-9], as do physicians in independent practice [10].

The findings of a single-centre study by Landrigan and colleagues of 20 interns working in an adult intensive care unit (ICU) [11,12] are often cited as compelling evidence in favour of reducing resident duty hours [13]. This research used a randomized cross-over design to compare 16-hour duty periods (intervention schedule) with 30-hour duty periods (traditional schedule). Rates of errors and adverse event outcomes were obtained by multiple concurrent methods. This study found a higher rate of serious medical errors in the traditional schedule than in the intervention schedule (136.0 versus 100.1 per 1000 patient-days, *p* < 0.001). Importantly, the serious medical errors outcome included errors with the potential to cause harm. The definitive outcomes reported were preventable adverse events (harmful errors) and mortality. There were no differences between the intervention and traditional schedules with respect to harmful errors in the ICU (38.6 and 38.5 per 1000 patient-days, respectively, *p* = 0.91), and mortality was not significantly higher in the intervention schedule (12.7% versus 14.5% *p* = 0.55) [11]. On average, alertness was lower in the 30-hour duty period; however, in four (20%) interns, an indirect electroencephalogram (EEG) measure suggested lower alertness during the 16-hour duty period [12]. These sleep and alertness data raise questions about a number of factors, including the generalizability of the conclusion that “less is more” to all first-year residents (or other physician groups), the adequacy of the sample size studied, and the relevance of resident sleep and sleepiness to harmful medical errors.

These randomized controlled trial data, along with other health services data showing time-related improvement in patient outcomes, call into question the notion that reducing resident duty hours improves patient safety [14]. Apparent improvements in outcomes over time in before-and-after studies of duty hours and other “safety interventions” [15,16] may be explained by other factors, including secular trends showing improvement in hospitals with and without residents [17]. As well, these studies may, in fact, show only minimal change after the introduction of duty hour regulations [18].

These data, summarized in the Institute of Medicine (IOM) report, suggest that, overall, resident duty hour reduction does not improve – nor does it worsen – meaningful patient safety and quality outcomes [19]. The IOM report states that “patient safety is affected by many factors and the research data available did not make it possible for the committee to assess the current level of all risks to patients *or the degree to which fatigued residents contribute to patient harm*” [19] (emphasis added). Four explanations for this apparent contradiction of public expectation are explored below.

### [1] Resident fatigue is a minor determinant of harmful errors

In the discourse on harmful medical errors, resident fatigue is frequently “implicated” as a significant causal factor [20-22]. Here we suggest that the relative contribution of fatigue to medical errors may be overstated, and that studies reporting harmful and other errors need to account for the duration of clinical exposure.

Evaluating the relative contribution of fatigue to significant medical error is challenging [19]. The ubiquitous, ill-defined notion of “fatigue” may be used as a proxy for other more specific individual- and system-level factors, including limited experience, limited content or patient-specific knowledge, high workload, and inadequate supervision. Studies focused primarily on these factors report that they are more frequently associated with medical errors than is fatigue [23-28]. Notable examples where fatigue has displaced discussion and recognition of other more important factors include the Libby Zion case, in which trainee experience, seniority, and supervision [29] were highlighted but subsequently downplayed, as well as Landrigan and colleagues’ landmark study of newly graduated physicians practicing in tertiary-quarternary adult ICUs [11,12].

A second factor is the exposure effect associated with working longer hours. It is reasonable to expect that individuals who work longer hours will observe or experience a greater number of harmful and other medical errors than those who work shorter hours, simply by virtue of their longer exposure to clinical situations. To date, this “exposure effect” has received limited attention in the literature [30,31]. Studies describing associations between self-reported physician burnout and/or depression and both longer duty hours and medical errors also overlook the effect of clinical exposure on these potentially correlated outcomes [24,32]. Uncritical acceptance of the results of these studies by clinicians and the public further perpetuate the notion that “long shifts” equate with “bad care.” In turn, this may fuel the demand for reform and shift focus and resources from other, more effective, safety mechanisms.

### [2] Resident duty hours are a minor component of resident fatigue

*If* we accept that the relative contribution of fatigue to harmful and other medical errors is significant, then the contribution of duty hours to resident fatigue warrants closer consideration. The origins of resident fatigue are multi-factorial [19]. Attributing fatigue mainly to hours of continuous duty and total duty hours is likely to be an over-simplification that overlooks workload, circadian rhythm disruption, tolerance of sleep loss, and other sleep-related factors [33].

Workload during the duty period (both on-call and during regular days) is an important source of resident fatigue. Workload varies significantly between rotations, specialities, and duty periods, and it is associated with reduced opportunities for on-call sleep [6,7,9]. At best, workload is independent of duty hour reduction. However, after duty hour reduction, workload-associated fatigue may be increased if the same work is compressed into fewer hours, and low workload rotations may be transformed into high workload rotations.

The degree of fatigue experienced by residents is influenced by factors such as disruption of the circadian rhythm and their individual tolerance of sleep loss. Working at night disrupts the circadian rhythm in physicians [8,34], nurses [35], and other shift workers [36]. Consequently it may be difficult to separate the effects of prolonged wakefulness or prolonged shift duration from those of shorter overnight work periods.

The role of personal preferences and tolerances [37] in the genesis of resident fatigue (sleep deprivation) also warrants consideration. An increased number of opportunities to sleep arising from duty hour reduction may not be paralleled by similar increases in the hours of actual sleep. In Landrigan and colleagues’ research, each hour of duty hour reduction was associated with only 20 minutes of increased sleep [11,12]. Other factors, including parenting, other family commitments, financial pressures, and educational requirements will also contribute to resident fatigue and burnout [38-40].

### [3] There are adverse consequences of reducing resident duty hours

If one accepts that the available laboratory and observational data indicate that resident fatigue influences patient safety outcomes, then it is still reasonable to ask whether the reduction of resident hours might nonetheless have harmful effects. The question then arises: “What factors counterbalance the beneficial effects of reduced resident fatigue?” One commonly articulated factor is lack of continuity, mediated through both reduced direct contact with patients and increased frequency of handovers [41-44]. Others include a shift work mentality [29,45], reduced resident supervision by responsible physicians resulting from reduced supervisor–trainee contact [6,46], and the cumulative effect of compromised education leading to physicians being inadequately prepared for practice in the real world [47-50].

Hospitalized patients are complex [51], and economic and other pressures encourage shorter lengths of stay in hospital [52]. Consequently, the need for health care providers to rapidly know and understand, appropriately investigate, provide optimal treatment, and effectively transfer the care of patients are all fundamental aspects of modern health care. This requires continuity of care. Continuity may originate from individual providers or from health care teams. Continuity operates across three domains:

Informational continuity – the use of information on past events and personal circumstances to make current care appropriate for each individual

1. Management continuity – a consistent and coherent approach to the management of a health condition that is responsive to a patient’s changing needs

2. Relational continuity – an ongoing therapeutic relationship between a patient and one or more providers (Haggerty and colleagues, 2003) [53]

Each, and all, domains of continuity may be threatened by duty hour reduction.

There are a number of ways in which duty hour reduction can compromise continuity: increasing the number of handovers; reducing the duration of clinical exposure to patients; increasing the intervals between exposure to patients; and reducing the proportion of available time for residents to interact and become familiar with individual patients and interact with other members of the health care team [54]. Physicians who are less familiar with their patients may make less-informed clinical decisions or delay decisions [55], or they may compensate for their lack of familiarity by ordering more tests [56]. In turn, these actions may undermine the quality and outcomes of care, as has been suggested in studies showing harm associated with care transitions [57] or duty hour reduction [55,58].

Despite the ease and frequency with which potential adverse consequences of long resident duty hours for patient safety have previously been articulated, separation of fatigue-related from continuity-related errors is inherently problematic, and this difficulty is compounded by the multidisciplinary and overlapping nature of health care teams. We suggest that the best evidence for the existence of these counterbalancing factors is the lack of improvement in meaningful patient outcomes associated with resident duty hour reduction. Irrespective of duty hours, continued efforts to improve the nature and quality of communication within concurrent multidisciplinary teams and at points of care transition remain an important area for patient safety [41,44,59].

### [4] Residents are of limited immediate consequence to patient safety

Residents are recent graduates, are explicitly acknowledged as trainees, require supervision, and are required to attend formal education sessions, complete informal requirements, and pass exit examinations before entering into independent practice. As such, one could argue that residents could pose a potential threat to the provision of optimal care. Conversely, appropriate resident training is required to sustain the number and quality of physicians in independent practice to ensure the safety of tomorrow’s patients.

The value of resident work has been expressed in a variety of ways: as a financial benefit [60], as a way of fulfilling the need to train doctors to care for future patients [49], and as the potential for residents to increase patient safety [19]. However, it is worth noting that some resident work can be successfully completed by others or can be significantly reduced through the use of health care technology. This suggests that residents may not be “essential” elements of care, something that is consistent with their role as trainees [6,19,61-63] and supports the notion that residents have limited ability to either add to or detract from patient safety.

## Other considerations

Several additional factors warrant consideration. The first is the nature of evaluations performed to date. Because these evaluations do not demonstrate clinically significant relationships between resident duty hours and patient safety, one may question the relevance of the studies that have been done. Future studies should evaluate a wider range of duty hours and include both short-term cross-sectional and longer-term system-level outcomes. The use of concurrent assessment of multiple domains (i.e., workload, fatigue, educational opportunity and outcome, and patient safety) will enable consideration of the relative impact of resident duty hours on each of these important domains.

Second, duty hour regulations usually describe maximum duty hours either for continuous duty, or for a certain period, or both [52]. The distinction between regulation and real-world practice is fundamental. If practice does not reflect regulatory change, then inferences linking changes in patient safety to changes in resident duty hours are moot [14,18]. Third, the impact of the local safety culture and professionalism warrants more rigorous evaluation as a potential factor mitigating patient safety following duty hour reduction [64,65]. Finally, the impact of fatigue tolerance, personal motivations, and evolving expectations and standards of care [66] will change the landscape against which the relationship between resident duty hours and patient safety is evaluated. Ongoing assessment is therefore needed.

## Conclusion

An increasing body of evidence undermines the assumption that long duty hours for residents compromise patient safety and quality of care. Conversely, the evidence that shorter duty hours compromise patient safety is weak. Delinking the association of duty hours, fatigue, and compromised patient safety is important beyond providing clarification of the basis for a socially desired change. The possible and probable reasons that resident schedule changes have not influenced important patient safety outcomes are many, and include the limited relevance of fatigue to the creation of harmful errors, the modest contribution of duty hours to the overall burden of fatigue, the fact that any beneficial effects of duty hour reduction are counterbalanced by adverse effects, and the fact that, as trainees in a complex system, residents are of limited relevance to patient safety. While disentanglement of these issues is desirable, the current literature is limited. Greater understanding will enable pre-emptive mitigation and optimization of a complex system that all seek to improve.

## Authors’ contributions

Both authors contributed to the concept, drafts, and revisions of this manuscript. Both approved the final version.

## Competing interests

The authors declare that they have no competing interests.

## Declarations

Resources and secretariat support for this project was provided by the Royal College.

This article has been published as part of *BMC Medical Education* Volume 14 Supplement 1, 2014: Resident duty hours across borders: an international perspective. The full contents of the supplement are available online at http://www.biomedcentral.com/bmcmededuc/supplements/14/S1. This article was submitted and peer reviewed in 2012. Final acceptance for publication as part of this supplement was in 2014.

Publication of this supplement was supported and funded by the Royal College of Physicians and Surgeons of Canada. The funding agency played no role in the design, in the collection, analysis and interpretation of data; in the writing of the manuscripts; and in the decision to submit the manuscript for publication. The articles have been through the journal's standard peer review process for supplements. The Supplement Editors declare that they have no competing interests.
